# Feasibility of early digital health rehabilitation after cardiac surgery in the elderly: a qualitative study

**DOI:** 10.1186/s12913-024-10601-3

**Published:** 2024-01-22

**Authors:** Bente Skovsby Toft, Lotte Ørneborg Rodkjær, Lotte Sørensen, Marianne Rørbæk Saugbjerg, Hilary Louise Bekker, Ivy Susanne Modrau

**Affiliations:** 1 Research Centre for Patient Involvement, Aarhus University Hospital, Aarhus, Denmark; 2 Department of Clinical Medicine, Cardiothoracic Surgery, Aarhus University, Aarhus, Denmark; 3 Department of Infectious Diseases, Aarhus University Hospital, Aarhus, Denmark; 4 Department of Public Health, Aarhus University, Aarhus, Denmark; 5 Department of Physiotherapy and Occupational Therapy, Aarhus University Hospital, Aarhus, Denmark; 6 Department of Cardiothoracic and Vascular Surgery, Aarhus University Hospital, Aarhus, Denmark; 7 Leeds Unit of Complex Intervention Development (@LUCID_Leeds), School of Medicine, University of Leeds, Leeds, UK; 8 Department of Clinical Medicine, Cardiothoracic Surgery, Aarhus University Hospital, Palle Juul-Jensens Boulevard 99, Aarhus, 8200 Aarhus N Denmark

**Keywords:** Qualitative research, Interview, Cardiac rehabilitation, Physical activity, Mobile health, Frail elderly

## Abstract

**Background:**

Increasing numbers of elderly patients experience prolonged decreased functional capacity and impaired quality of life after seemingly successful cardiac surgery. After discharge from hospital, these patients experience a substantial gap in care until centre-based cardiac rehabilitation commences. They may benefit from immediate coaching by means of mobile health technology to overcome psychological and physiological barriers to physical activity. The aim of this study was to explore the usability, acceptability, and relevance of a mobile health application designed to support remote exercise-based cardiac rehabilitation of elderly patients early after cardiac surgery from the perspective of patients, their relatives, and physiotherapists.

**Methods:**

We adapted a home-based mobile health application for use by elderly patients early after cardiac surgery. Semi-structured dyadic interviews were conducted with a purposive sample of patients (*n* = 9), their spouses (*n* = 5), and physiotherapists (*n* = 2) following two weeks of the intervention. The transcribed interviews were analysed thematically.

**Results:**

Three themes were identified: 1) creating an individual fit by tailoring the intervention; 2) prioritizing communication and collaboration; and 3) interacting with the mobile health application. Overall, the findings indicate that the mobile health intervention has the potential to promote engagement, responsibility, and motivation among elderly patients to exercise early after surgery. However, the intervention can also be a burden on patients and their relatives when roles and responsibilities are unclear.

**Conclusion:**

The mobile health intervention showed potential to bridge the intervention gap after cardiac surgery, as well as in fostering engagement, responsibility, and motivation for physical activity among elderly individuals. Nevertheless, our findings emphasize the necessity of tailoring the intervention to accommodate individual vulnerabilities and capabilities. The intervention may be improved by addressing a number of organizational and communicational issues. Adaptions should be made according to the barriers and facilitators identified in this study prior to testing the effectiveness of the intervention on a larger scale. Future research should focus on the implementation of a hybrid design that supplements or complements face-to-face and centre-based cardiac rehabilitation.

**Trial registration:**

Danish Data Protection Agency, Central Denmark Region (1–16-02–193-22, 11 August 2022).

**Supplementary Information:**

The online version contains supplementary material available at 10.1186/s12913-024-10601-3.

## Background

An increasing number of elderly patients with multiple chronic conditions experience prolonged decreased functional capacity and impaired quality of life after seemingly successful heart surgery [1]. Exercise plays a pivotal role in cardiac rehabilitation (CR) and has proven efficacy in preventing and/or improving sarcopenia [2]. Furthermore, it contributes to enhancing physical capacity and independence in daily activities [3, 4]. Participation in physical activity during the early months after cardiac surgery is crucial for enhancing future health and well-being, particularly among elderly patients who may derive even greater benefits from exercise interventions compared to their younger counterparts [5].

In Denmark, standard care after cardiac surgery entails in-hospital mobilization, advising a gradual increase in physical activity levels after discharge, and referral to CR. Centre-based physical training is typically initiated approximately six weeks after surgery, aligning with sternal healing [6]. In the interim period between discharge and the start of centre-based CR, there is a gap in physical activity support between discharge and the start of centre-based cardiac rehabilitation training. Engaging in early post-cardiac surgery exercise significantly enhances future health, particularly for older patients, and is considered both effective and safe [7]. Mobile health (mHealth) has the potential to provide coaching and support for elderly patients in overcoming physical activity barriers [8, 9]. It may be favoured by patients for accessibility and flexibility [4]. However, several barriers may limit the application of mHealth in the elderly. These encompass psychosocial and physiological factors [5, 8, 9], limited familiarity with emerging technology, slow adaption, and hesitancy or apprehension about using it [10, 11]. Therefore, it is crucial to ensure that the mHealth application (app) is feasible and acceptable among elderly patients in terms of perceived usefulness and ease of use prior to implementation [5]. The use of mobile technology among the elderly population is rapidly increasing. In Denmark, smartphones are used by 47% of the Danish population aged 75–89 years [12].

In Denmark, the Icura® Activity technology by Icura Aps, Copenhagen has emerged as an mHealth solution designed to enhance rehabilitation following orthopaedic surgery [13]. It has proven to be effective in facilitating home-based exercise, and has been optimized for elderly users [14]. The technology offers personalized exercise programmes with visuals, wearable sensors to monitor physical activities, and a chat function via text messaging.

However, a research gap exists regarding the feasibility of using the Icura® Activity app for exercise intervention in elderly patients early after cardiac surgery. This evaluation is a crucial step before a randomized controlled trial that seeks to investigate the intervention’s efficacy in enhancing physical capacity and overall well-being after cardiac surgery. With this study, we want to ensure that the provision of this mHealth app intervention in the context of CR fits providers’ and users’ needs, resources, and competencies.

## Methods

This study aimed to explore the usability, acceptability, and relevance of an mHealth app designed to support remote exercise-based CR of elderly patients early after cardiac surgery from the perspectives of patients, their relatives, and physiotherapists.

This qualitative study employed semi-structured dyadic interviews and thematic analysis utilizing a hermeneutic methodology [15]. The framework for the assessment of acceptability informed the interview guide development, data collection, and analysis [16].

Conducted as part of a larger project, this pilot study serves as the developmental phase of a complex intervention (pre-phase 1) [17, 18], aiming to explore facilitators and barriers to successful intervention implementation prior to a planned randomized controlled trial.

### Setting, sampling, and participants

The study was conducted by a research team of physiotherapists, nurses, and cardiac surgeons at Aarhus University Hospital, Denmark.

A purposive critical case sample was selected by a senior cardiac surgeon (ISM) among elderly patients (age ≥ 70) with chronic disease and scheduled for elective open-heart surgery. Patients were included after giving written informed consent to test the mHealth app for two weeks following discharge, and subsequently to be interviewed together with their relatives about their perceptions and experiences. Physiotherapists involved in delivering the mHealth app intervention were interviewed to gain insights into their perspectives on the intervention. For this study, we predetermined a practical sample size of 8–12 participants, not aiming for data saturation. Instead, we focused on obtaining detailed data from a limited number of participants to minimize potential flaws in intervention delivery and reception.

### Trial registration

The study was registered with the Danish Data Protection Agency, Central Denmark Region (1–16-02–193-22) on 11 August 2022. The Research Ethics Committee waived study approval as interview studies are exempt from registration under Danish law (Denmark Committees on Health Research Ethics, §14, 2). The principles of the Declaration of Helsinki were followed [19], and written informed consent was obtained from all participants.

### The intervention

We employed the Icura® Activity technology, which includes a sensor based on accelerometry and a smartphone app that provides a training programme with exercise videos. Feedback was provided to the physiotherapists through a web-based platform showing planned and performed activities with the possibility for them to change the programme and interact with the patients using text messages. The exercise intervention, which commenced immediately after discharge, involved a daily programme comprising a specific number of steps, sit-to-stand movements, knee extensions, standing leg curls, pelvic lifts, and shoulder flexions. This programme was tailored to suit each patient, with modifications in both step count and exercise routine during the intervention period. Physiotherapist oversight took place 2–3 times weekly, reinforced by personalized feedback communicated through text messages. Phone calls were added as an adjustment of the intervention to better address the patients’ needs following an analysis of the initial patients (see Additional file 1).

### Data collection

Face-to-face in-person qualitative semi-structured interviews were carried out with patients undergoing the intervention (*n* = 9), their relatives (*n* = 5). Four of the interviews were conducted individual due to the absence of relatives. The two physiotherapists responsible for delivering the intervention (*n* = 2) were interviewed together. The interviews, audio recorded in a hospital meeting room, aimed for a conversational tone, allowing the informants to freely share their views and experiences. The participants could supplement each other and draw forth responses that might not otherwise have been recognized or remembered.

To understand participants’ responses to and interactions with the intervention, we used a theoretical topic guide (see Additional file 2). Derived from the theoretical framework of acceptability, this guide ensured interview consistency. It covered factors like ethicality, affective attitude, burden, effectiveness, self-efficacy, and intervention coherence [16]. Moreover, questions regarding feasibility aimed to explore the suitability and acceptability of the intervention among the participants. All interviews were conducted by the first author (BST), a physiotherapist and health service researcher experienced in rehabilitation and qualitative methods, without prior acquaintance with the participants. The initial interview served as a pilot without necessitating guide changes, and its data were included in the study.

### Data analysis

Data analysis was performed using analysis guided by Braun and Clarke’s six-phases [15]. In the first phase, interviews were transcribed verbatim and read multiple times to gain a comprehensive understanding of the data and identify both latent and manifest content. In the second phase, initial codes were generated systematically for interesting features of the data. The NVivo® software, Lumivero, Denver, USA, was used to organize the codes. In the third phase, an open and reflective method was used to collate codes and identify patterns of shared meaning. The first and second phases of the analysis were conducted by the first author; in the third phase, BST and MRS gathered all data relevant to potential themes and the co-authors reviewed the findings for accuracy. Central sub-themes and themes were identified based on the collated codes and reviewed by the co-authors to ensure they accurately reflected the data. In the fourth phase, a thematic map was generated to visually represent the codes and their relationship to the findings. In the fifth phase, definitions and names for each theme were refined by all authors, with any discrepancies resolved prior to developing the themes. Finally, in the sixth phase, extract examples were selected based on their relevance to the research question [15]. Member checking was not performed as it is not consistent with the methodology of thematic analysis.

In the results, we removed any repeated and unnecessary words from the quotations to make the sentences complete and understandable while still retaining their meaning. The study followed the standard criteria for reporting qualitative research [20].

## Results

Out of the 15 patients enrolled (13 men, 2 women), six did not complete the study intervention. The reasons for non-completion included discharge without equipment (*n* = 1), withdrawal of consent (*n* = 2), post-surgical complications leading to extended hospital stay (*n* = 2), and one patient’s unfortunate passing. In total, nine participants successfully engaged in both the intervention and subsequent interviews. All participants’ characteristics are detailed in Table 1. Five patients had joint interviews with a relative, while the other four were interviewed alone. These sessions took place between September 2022 and March 2023, averaging 34 min each (range: 25–43 min).

**Table 1:** Characteristics of patient participants

Demographics and type of surgery	Patient participants (*n* = 9)
Age, median (range)	75 (73–79)
Male	8 (89%)
Danish ethnic origin	9 (100%)
Occupation: retired	9 (100%)
Marital status:	
Married/Co-habiting	6 (67%)
Single/Living alone	3 (33%)
Education:	
< 10 years/short	2 (22%)
10–12 years/medium	6 (67%)
> 12 years/long	1 (11%)
Type of surgery:	
Coronary artery bypass graft	2 (22%)
Heart valve surgery	6 (67%)
Both (coronary artery bypass graft and heart valve surgery)	1 (11%)
Prolonged hospitalization	5 (55%)
Relative attending interview:	5 (55%)
Spouse	3
Brother	1
Daughter	1

We conducted an interview with two female physiotherapists, one of whom held a master’s degree. They possessed 19 and seven years of work experience, with over 10 and 3.5 years of specialization in CR respectively. The interview with the physiotherapists took place in March 2023, and lasted 65 min.

### Interview findings

Data were analysed and categorized into three themes: 1) creating an individual fit by tailoring the intervention; 2) prioritizing communication and collaboration; and 3) interacting with the mHealth application. Themes and sub-themes are presented in Table 2, with more illustrative quotes available in Additional file 3.

**Table 2:** Overview of themes and sub-themes

Theme 1	Theme 2	Theme 3
Creating an individual fit by tailoring the intervention	Prioritizing communication and collaboration	Interacting with the mobile health application
**Sub-themes**		
1a: Unilateral focus on exercise and physical factors	2a: Sufficient support and follow-up	3a: Using technology and monitoring
1b: Acknowledging a demanding start	2b: Balancing obligations and responsibilities	3b: Timely delivery and organization of intervention
1c: Individual adaptions responding to users’ preferences and capacities	2c: Involvement and roles of relatives	3c: The value of closing an intervention gap

### Theme 1: Creating an individual fit by tailoring the intervention

#### 1a: Unilateral focus on exercise and physical factors

Perceived from the patients’ standpoint, the intervention primarily emphasized physical activity and exercise, enhancing their awareness of and ability to engage in regular physical activity, pushing them to go beyond everyday tasks. Tracking exercise completed and distances walked allowed them to monitor progress and goal attainment. However, some patients and relatives criticized the intervention for neglecting well-being and psychosocial aspects. They requested the physiotherapists to address emotional and motivational aspects like mood, energy, and insecurity.

*“It is a limitation [that the intervention did] not [address] the psychological issues, which relatives ought to know about.” R4*

The physiotherapists emphasized the importance of addressing the patients’ physical activity level and acknowledged that time and opportunities to discuss patients’ experiences and concerns were limited.

#### 1b: Acknowledging a demanding start

Delays in starting exercise were mainly due to prolonged hospital stays because of post-operative complications. Starting the programme after discharge required strong self-discipline. Reduced strength, dizziness, pain, and breathlessness exacerbated the situation, making physical activity more challenging. Fatigue and depression were major barriers to start-up, and the exercise programme’s workload added to the burden.

*“I don’t think the programme did any good. When I took it home, my body said no, and it was a kind of defeat that I could not do these exercises in the programme, and it caused some frustration.” P6*

Additionally, mental and emotional elements such as anxiety, resignation, lowered mood, anger, irritability, and scepticism were mentioned as influencing patients’ physical activity efforts – but also their engagement in supportive social relationships.

The physiotherapists acknowledged the demands of initiating exercise but found it difficult to help the patients overcome barriers related to physical and mental difficulties within the current intervention design. They realized that delivering and supporting the mHealth intervention required additional physiotherapist resources compared to usual treatment.

#### 1c: Individual adaptions responding to users’ preferences and capacities

Some patients tried to adapt the programme to their needs. Others waited for the physiotherapists to make adjustments, which were generally welcomed despite not always being a perfect fit. However, the intervention did not entirely match the diverse preferences, needs, skills, and capacities of all the patients and relatives. A more tailored approach can be expected to heighten its effectiveness.

*“I did 60 [repetitions], then 30 and then the other leg…that’s how I increased [the number of exercises]. I have done more than 326 [repetitions]…but that was because it was the only exercise I could really do.” P9*

Patients’ prior experiences and activity preferences varied, leading to diverse responses to the intervention. Some felt empowered by the programme, overcoming barriers and taking responsibility for their physical activity. Some managed to improve the programme fit by altering repetitions or dividing exercises into multiple sessions, enjoyed exercising enthusiastically, and were pleasantly surprised by their improved well-being and mood. Conversely, some experienced physical discomfort and discouragement and felt disheartened when they were unable to keep up with the programme. Some of them attempted to seek support from the physiotherapist to match the workload to their capacities, while others refrained from seeking support. The majority of the patients preferred doing routine tasks like housekeeping, gardening, walking or cycling.

The physiotherapists reflected on better integration and prioritization of everyday tasks. They perceived it as challenging to make individual adaptions primarily based on sensor feedback with only limited knowledge about patients’ preferences and capacities. The patients, relatives, and physiotherapists all emphasized the importance of having an alternative to mHealth for vulnerable patients who require a more comprehensive rehabilitation plan.

### Theme 2: Prioritizing communication and collaboration

#### 2a: Sufficient support and follow-up

The text messages from the physiotherapists with reminders and encouraging comments were well received by the patients. They viewed these as expressions of interest and engagement, providing security and motivation.

*“Somehow, I felt that someone was on the other end saying, ‘It looks fine, what you are doing,’ registering and following what I did. That was nice.” P4*

Despite being explicitly informed of it before discharge, participants rarely utilized the option to reach out to the physiotherapists via text message or phone call. Contact criteria remained unclear, as the physiotherapists expected two-way communication while patients often preferred to manage their activity independently. To address this missed communication with some patients, we added one or more phone calls to provide follow-up to those who refrained from seeking support. The subsequent dialogues emphasized the vital necessity for personalized contact, covering well-being, programme execution, and daily concerns. The physiotherapists emphasized the importance of allocating ample time to addressing individual needs and concerns to ensure effective implementation and intervention quality.

#### 2b: Balancing obligations and responsibilities

Among those capable of engaging in the exercises and tasks, the intervention was perceived as motivating and added to their sense of responsibility. Others regarded it as an unwanted duty and felt burdened by high expectations for rigorous physical activity, leading to discouragement. Having consented to test the mHealth app intervention made them feel obligated to complete the programme. The exercise programme was not always well matched to patients’ abilities or preferences.

*“I could only manage a few repetitions of the exercise before feeling exhausted, and then the counter indicated that I had to complete another 30 repetitions.” P14*

Being unable to complete the programme added to a sense of defeat and made some give up and stop using the technology. Sharing responsibility with others eased the burden.

The challenge for the physiotherapists was to facilitate physical activity without making patients who were incapable of completing the full exercise programme feel inadequate or guilty.

#### 2c: Involvement and roles of relatives

All the relatives wanted increased involvement, for example by being present for the decision about enrolment, the introduction of the technology, and the presentation of the exercise programme. They were viewed as a resource by the patients. However, they lacked knowledge about how to support initiation of the exercise programme and adherence to it when difficulties or complications arose. It was challenging for the relatives to balance their behaviour between pressure and support without impacting on their relationship with their loved ones in a negative way. Disagreement between patients and relatives about physical activity efforts could result in conflicts and frustrations for both.

*“I am sure I could easily have completed it [the programme] without having a physiotherapist as back-up.” (P12) (The relative adds:) “I don’t think you would have been able to, if the physiotherapist had not been there during the first weeks.” R12*

The role of an engaged relative could be demanding. Some sought more information, guidance, and professional support, while others engaged their network for assistance with the technology, adapting the programme or deciding to discontinue exercise.

The physiotherapists perceived the relatives as an important resource for the patients but did not involve them systematically.

### Theme 3: Interacting with the mobile health application

#### 3a: Using technology and monitoring

Operating the smartphone, app, and text messages proved to be straightforward for the majority of patients. Even those who were initially worried by the technology were pleasantly surprised by their proficiency, with only a few needing assistance initially. Patients particularly appreciated the visual exercise guides, the monitoring features, and the reassurance provided by the option to communicate with their physiotherapist.

Some patients found monitoring their exercise gave them enjoyment, prevented boredom, and provided a sense of security, encouragement, and collaboration. Competitive patients used the monitoring to compete with themselves by following their progressions and reaching their explicit goals. Others perceived the monitoring of exercises as a way of having their exercise efforts measured and controlled as well as having their incompetence exposed. Two participants mistakenly took the sensor to be for online surveillance of physiological parameters, e.g. blood pressure during activity.

Initially, the physiotherapists did not consider the elderly to be a suitable target group for an mHealth app intervention and anticipated potential barriers to adoption.

*“In the group of elderly we want to reach, there may be some who feel like strangers to technology and have difficulty in using it on their own. If they [the patients] can make it [the technology] work, I think it would be very good for some of them.” PT*

The physiotherapists viewed the monitoring as an exercise diary that illustrated programme suitability and demonstrated the patients’ self-management abilities. However, they acknowledged the risk of overlooking concerns and challenges for the patients that were not captured in the data.

One patient found the technology troublesome and discontinued using it. Another quit the programme because it was too burdensome.

#### 3b: Timely delivery and organization of intervention

Identifying the ideal time frame for equipment delivery and instruction before discharge presented practical challenges and was difficult to coordinate with treatment and care activities for the patient. In some cases, last-minute discharges prevented relatives from being invited to or attending the session giving instructions. Receiving, understanding, and remembering information and making decisions about enrolment were difficult for some patients close to the time of surgery.

*“I did not pay much attention to the instructions because the surgery filled me up. Actually, it was in my head for the last three weeks…because I was pretty sure that I would succumb, and I guess I thought, ‘Well, just let me get it over with.’ I probably didn’t listen as I should have done.” P15*

The amount of information was perceived as being overwhelming and caused a sense of information overload in some of the patients. The time allocated for the instructions and repetitions was insufficient from the perspective of the physiotherapists and did not meet the needs of participants with reduced short-term memory or attention problems. One physiotherapist found the organization and procedures suboptimal and wondered if a specialized university hospital was the right setting for delivering the intervention.

#### 3c: The value of closing an intervention gap

The aim of becoming active early after surgery was consistent with the values of the patients, the relatives, and the physiotherapists. The intervention was considered to be effective, meaningful, encouraging, and motivating and made a positive difference for most patient and relatives.

*“The exercise would not have been systematic if it had not been for the programme. It would have been random. A programme like this commits me to others. I think that is the value of it.” P4*

The flexibility was appreciated, as it made the exercise easy to fit into everyday life, and the fact that no transport was required reduced the risk of patients feeling they were being a nuisance to others. Some patients considered the intervention to be a helping hand to get started, whereas others only participated because it was a research project. The intervention inspired some to continue exercising and attend the centre-based CR in the municipality. Others were more reluctant and wanted to talk to the rehabilitation team before deciding to enrol. Despite their different experiences of the intervention, all participants and their relatives would recommend the intervention to others.

The physiotherapists considered the intervention to be relevant to prevent fatigue, inactivity, and loss of muscle in elderly patients. They were not convinced that the mHealth app was suitable for all patients or better than their usual guidance to patients on being physical activity after surgery. However, they could see the potential of the mHealth app as a complementary method to supplement existing CR.

In summary, various facilitators and barriers are relevant to consider in the delivery of the intervention (see Table 3).

**Table 3:** Facilitators and barriers related to acceptability, usability, and relevance of the mobile health application intervention as perceived by the providers and the users

	**Facilitators**	**Barriers**
**Usability**	User-friendly device Visuals and interactive functionalities Performance tracking and assessment A structured and personalized programme Sharing and discussion of experiences Prompt and ample support Text messaging capability Subsequent phone consultations Self-management abilities	Technological difficulties Inadequate capabilities and apprehensions^b^ Insufficient guidance or overwhelming information Aversion to tasks or challenging assignments Unacknowledged workload pressures Overemphasis on physical activities Deficiencies in self-discipline^b^
**Acceptability**	Tailored to individual needs and preferences Monitoring and achieving successful outcomes^b^ Recognition and appreciation of personal efforts^b^ Pleasure derived from physical activity^b^ A feeling of empowerment^b^ Sharing/joint responsibility^b^ Availability for in-person conversations Ensuring prompt and timely delivery Punctual delivery Well-defined/manual for delivery procedures^a^	Coping with vulnerabilities experienced such as depression, fatigue, and anxiety Experiencing a sense of obligation and pressure^b^ Absence of adaptations personalized to individual needs Uncertainty regarding expectations, responsibilities, and roles Inadequate involvement of and support from family members Struggles and conflicts between patients and their relatives Constraints on time during intervention delivery Limited opportunities to address concerns related to well-being, motivation, and emotional aspects Lack of cross-sectoral collaboration^a^
**Relevance**	Addressing the needs, preferences, and capabilities of both patients and their family members Sustaining adaptability in daily routines^b^ Convenient home-based approach (no transport required)^b^ Promoting awareness of and motivation for regular physical activity Commencing exercise promptly after surgery Reducing sedentary periods and enhancing exercise engagement Incorporating meaningful activities Cultivating positive interest and anticipating beneficial outcomes Feasible as a complementary intervention	Exclusive emphasis on physical activity Lack of insights into psychosocial, emotional, and existential aspects Insufficient social interaction^b^ Extended hospital stays, post-surgery fatigue, and complications Diminished energy, initiative, and motivation Requirement for personalized in-person physiotherapy Lack of coordination of delivery with concurrent activities and tasks Lack of access to technology after end of intervention

Only addressed by: ^a^providers or ^b^users

## Discussion

This study set out to explore the usability, acceptability, and relevance of an app to promote physical activity among elderly patients early after cardiac surgery. Overall, our findings validate the applicability of the mHealth intervention in this context. However, it is crucial to consider various factors including facilitators and barriers for the upcoming clinical trial.

### Usability

We found the mHealth solution had the potential to increase physical activity early after cardiac surgery among elderly patients, making it relevant to offer to this group. Usability was influenced by each person’s subjective experiences and capabilities, hence healthcare providers and patients may have different perspectives and barriers to using mHealth [21] as well as different levels of competencies to use it [22]. Our findings are in line with a prior study describing the willingness of elderly adults to embrace new technology if the potential benefits outweigh the effort required [11]. Usability was significantly affected by each individual’s subjective experiences and abilities. Our findings are consistent with a systematic review which found that the conversation about CR taking place at an inappropriate time during the in-patient stay may result in information overload becoming a barrier for the intervention [23]. Therefore, we agree that the focus should be on achieving the right balance between information and support to avoid either overwhelming or inadequate information [1].

With these new insights, the usability in the upcoming clinical trial will be improved by the development of an intervention delivery manual and providing written information material to patients, as well as offering prompt and ample support.

### Acceptability

Our study revealed that inter-individual differences and barriers to initiating early physical exercise influenced acceptance of the mHealth programme. Therefore, tailoring the intervention to meet the various expectations and needs of individual patients and their relatives seems crucial. Recovering from heart surgery is often a dynamic, complex process. The requirement we identified for an individualized approach concurs with existing qualitative research on patients’ perspectives on recovery after heart valve surgery, which suggests that the need to address physical, mental, and existential challenges should be met systematically and early after surgery [1]. An individualized approach that incorporates feedback and self-monitoring can enhance motivation, physical activity level, and self-autonomy [24] and has the potential to increase the effectiveness of mHealth interventions [25]. It may help overcome barriers and prevent problems of low uptake and adherence to centre-based CR in patients after open-heart surgery [26]. Overcoming perceived barriers to the use of mHealth interventions among elderly patients may require social support from family and healthcare providers [27]. The patients, relatives, and physiotherapists in this study emphasized communication and collaboration as being most important to support the relatives’ level of involvement without imposing unsustainable burdens and unwanted roles. Findings suggest patients would benefit from the implementation addressing both emotional needs as physical exercise. Enabling physiotherapists to address emotional needs may support adherence to post-surgical rehabilitation. To enhance acceptability in the future delivery of the intervention, some structural changes may increase acceptability among physiotherapists by allocating sufficient time for dialogue and collaboration.

To enhance acceptability in the clinical trial that will follow, we suggest an even more finely tailored and individualized approach based on clear communication and close collaboration between patients, relatives, and physiotherapists, with shared responsibility for CR. To improve feasibility, organizational adjustments should be considered to enable continuous support and follow-up.

### Relevance

This mHealth intervention is a complex home-based intervention designed to bridge the gap until traditional centre-based CR starts. Evidence indicates the feasibility of engaging in physical activity shortly after cardiac surgery, even in the presence of reported pain and discomfort [28]. Furthermore, home-based CR carries a minimal risk of adverse effects [29]. This underscores the relevance of considering home-based CR either alone or in combination with centre-based CR to overcome some of the known barriers for traditional CR [30]. Non-participation in CR has been associated with factors such as older age and female gender [31], as well as having symptoms of depression or anxiety [32].

In Denmark, the uptake rate for CR among patients undergoing heart valve surgery has been found to be around 50%, with a lower rate among older age groups [33]. Moreover, the drop-out rates from CR are high, and patients have reported dissatisfaction with the content of interventions, which have been criticized for lacking a psychosocial element and the involvement of relatives [34]. However, a guided mHealth intervention for CR has proven to be effective, beneficial, and safe for elderly patients who had declined participation in traditional centre-based CR [35]. Our findings suggest that exercise-based mHealth interventions are most relevant in a hybrid design complemented by other methods of delivery, e.g. telephone counselling or face-to-face physiotherapy, especially for users with special needs or who are incapable of engaging with the technology.

To address the mental health needs, our upcoming trial will increase the focus of physiotherapists on integrating crucial emotional and social factors for post-surgery well-being. Furthermore, our plan involves enhancing interdisciplinary collaboration by involving nurses, general practitioner and, when needed, psychologists more liberally to address concerns related to anxiety and depression. We will strive to meet individual patients’ preferences and needs, incorporating gender and/or age differences, and improve the involvement of relatives.

### Strengths and limitations

This study provides evidence about using modern technology with a group of elderly patients testing a specific mHealth intervention. The dyadic interviews provided both individual and shared narratives from patients and relatives [36]. The hybrid deductive/inductive approach provided structure for data generation and analysis as well as in-depth understanding of the participants’ experiences of facilitators and barriers for the intervention [37].

A limitation is our relatively small sample, primarily consisting of Caucasian, married, well-educated males. Nevertheless, this distribution aligns with the current gender representation (20% female patients) in open cardiac surgery at our institution. Additionally, this exploratory pilot study may have been constrained by its two-week testing period within a planned six-week intervention. This shorter time frame might have generated data during the early post-operative recovery phase, where factors like fatigue and pain may be more pronounced [1].

### Patient involvement

This qualitative pilot study did not integrate public involvement due to its preliminary nature focusing on understanding the feasibility and initial insights of the intervention. However, integrating such involvement is planned as part of the forthcoming intervention trial and implementation phase.

### Future implications

In response to the findings of this study, we recommend some refinements to improve the content and delivery of the intervention before progressing to the next research phase:

- Develop a manual for delivering a tailored intervention including bio-psycho-social and existential factors and the involvement of relatives.
- Provide written patient information with key messages and processes.
- Be more specific about contacting physiotherapist in case of challenges.
- Plan proactive phone calls and support to patients and relatives, and continuously follow up.
- Collaborate with patients and relatives on designing the programme and adapting it to their capacities, preferences, and needs.
- Allocate sufficient resources to physiotherapist and identify the optimal time for delivery.
- Ensure interdisciplinary collaboration on issues related to anxiety and depression.
- Consider a flexible intervention composition, e.g. hybrid or complementary intervention.

Future research should focus on implementing the mHealth intervention as a part of an integrated patient pathway that supplements or complements face-to-face and centre-based CR. It is likely the development of a shared decision making intervention to enable physiotherapists to support people post-surgery to decide between different types of CR (mHealth, centre-based, hybrid approach) will be able to address patient and professional needs identified by our research, and enhance service delivery and patient outcomes [38].

## Conclusion

The present study demonstrates the usability, acceptability, and relevance of remote mHealth intervention early after cardiac surgery for elderly patients. This intervention shows promise in closing the intervention gap and fostering engagement, responsibility, and motivation for physical activity. Our findings highlight the necessity of tailoring the intervention to align with users’ needs and accommodate their distinct experiences. Tailoring of the intervention to meet the users’ needs and to address individual experiences of vulnerabilities and capabilities is essential. For the upcoming randomized controlled trial, we recommend refining the delivery of the mHealth intervention by developing a manual for providers, providing written information for patients and relatives, and collaborating with patients and relatives to design an individualized and flexible programme.

### Supplementary Information

**Additional file 1.** Standard Operating Procedure.

**Additional file 2.** Study topic guide for patient-relative and physiotherapist dyads.

**Additional file 3.** Quotations from patients (P), relatives (R), and physiotherapists (PT) illustrating themes and sub-themes.

## Data Availability

No datasets were generated or analysed during the current study.

## Abbreviations

App: Application
CR: Cardiac rehabilitation
mHealth: Mobile health
P: Patient
PT: Physiotherapist
R: Relative
