# Supplementary material for: Feasibility of early digital health rehabilitation after cardiac surgery in the elderly: a qualitative study
Source: BMC Health Serv Res. 2024 Jan 22;24:113. doi: 10.1186/s12913-024-10601-3 (PMC10801932; doi:10.1186/s12913-024-10601-3)

Standard operating procedure (SOP), Icura Activity technology

Inclusion

- Particularly vulnerable patients with age ≥70 years with comorbidity will be selected from patients scheduled for surgery (cardiac surgeon).

  On the day of surgery preparation (typically the day before surgery)
- Eligible patients will be informed about the study, invited to participate, and asked to sign a written informed consent if they are willing to participate (project nurse).
- The Short Physical Performance Battery test will be conducted (project physiotherapist).
- A brief introduction to the Icura Activity technology will be provided (project physiotherapist).
- The devices are provided.

During hospitalization

- Close to discharge, the Icura Activity technology will be set up for the patient with a default standard daily program used in this study. The program was adjusted to the patient's specific capacity if needed (project physiotherapist).

  Standard daily program
  - Steps: 2000
  - Sit-to-stand: 3x10
  - Knee extensions: 3x10 with each leg
  - Standing leg curls: 2x30 sec.
  - Pelvic lifts: 3x5
  - Shoulder flexions: 3x10

Intervention – early digital health rehabilitation

- 2-3 times per week, the web-based platform will be reviewed, and feedback will be provided to the patients on performance through the chat function of the Icura Activity technology or telephone calls (project physiotherapist).
- Encouraging comments are provided to increase adherence (project physiotherapist).
- Individual adjustments to the daily program will be made, and any questions received through the chat function from patients or relatives will be answered (project physiotherapist).
- If activity level are not maintained according to the program telephone follow-up is provided (project physiotherapist).
- During the intervention period, patients and relatives will have a contact telephone number and mail address for a project physiotherapist if they have any questions or challenges (project physiotherapist).

End of intervention:

- The Short Physical Performance Battery retest will be conducted (project physiotherapist).
- The devices are collected (project physiotherapist)
- Interviews are conducted (researcher)

Rapid cycle evaluation and adjustments half way through the study

- The Short Physical Performance Battery test was performed by project nurse instead of physiotherapist to enhance organization flexibility.
- Information and introduction to the Icura Activity technology during hospitalization were reduced due to patients feeling overwhelmed with information.
- A telephone call from a project physiotherapist shortly after discharge from the hospital was implemented to facilitate the initiation of the intervention and provide information when needed.
- The use of the chat function of the Icura Activity technology was decreased, and telephone calls were increased, as only few patients utilized the chat function.

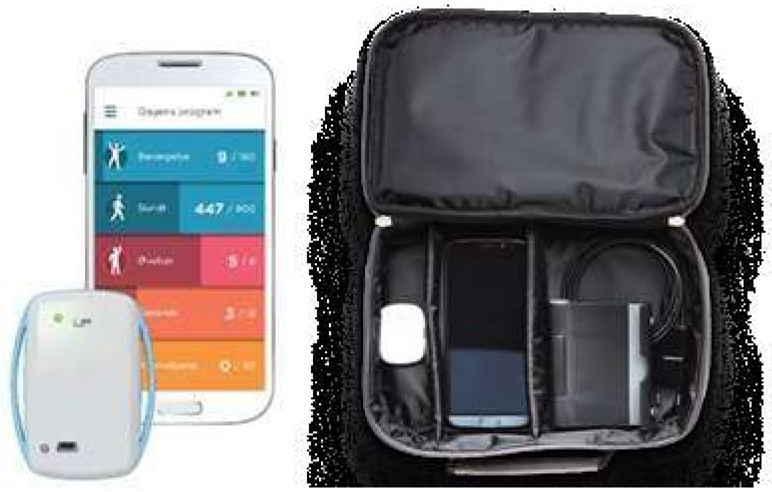

Supplement: Supplementary file 1 — Additional file 1. Standard Operating Procedure. [file 12913_2024_10601_MOESM1_ESM.pdf]
