# Supplementary material for: Feasibility of early digital health rehabilitation after cardiac surgery in the elderly: a qualitative study
Source: BMC Health Serv Res. 2024 Jan 22;24:113. doi: 10.1186/s12913-024-10601-3 (PMC10801932; doi:10.1186/s12913-024-10601-3)
Supplement: Supplementary file 2 — Additional file 2. Study topic guide for patient-relative and physiotherapist dyads. [file 12913_2024_10601_MOESM2_ESM.pdf]

Additional file 1: Study topic guide for patient-relative and physiotherapist dyads

Theoretical framework of acceptability	Definition	Topics	Examples of questions for patients/relatives	Examples of questions for physiotherapists
Ethicality	How the intervention fits personal values	• Considerations<br>• Meaningfulness<br>• Benefits and disadvantages	• What do you think about being asked to participate in the project?<br>• Which benefits and disadvantages did you consider?<br>• Why did you choose to participate?	• What do you think about the mHealth intervention?<br>• Did you have any concerns about engaging with it?<br>• What were your expectations?
Affective attitude	How the participant feels about the intervention prior to and after taking part	• Motivation<br>• Mood and energy<br>• Support	• How did you find participating?<br>• Did you experience any surprises (positive/negative)?<br>• Did it influence your energy and mood? How?	• How did you find delivering the intervention?<br>• Did you experience any surprises (positive/negative)?<br>• How were your engagement and relationship with the patient?
Burden	The extent to which the effort required to engage in the intervention was expected to be/experienced as a burden	• Prerequisites<br>• Efforts<br>• Challenges	• How did you find following the programme and using the app?<br>• What challenges and successes did you experience?<br>• How did you experience support from others?	• What did the intervention require from you?<br>• What changes would you suggest?<br>• How were your interactions and relationships with the patients?
Opportunity costs	The benefits, profits, or values that were expected to be/experienced as being given up to engage in the intervention	• Fit with everyday life<br>• Preferences<br>• Needs	• What do you think of exercising early after surgery?<br>• Did you have any particularly good or bad experiences?<br>• How well did it fit your everyday life?	• How do you think the app-based intervention suits the elderly cardiac patients?<br>• Do you have any concerns about using the mHealth intervention?<br>• Is the intervention consistent with good physiotherapeutic practice?
Perceived effectiveness	The extent to which the intervention was expected to achieve/experienced as achieving its purpose	• Outcomes<br>• Effectiveness<br>• Importance	• What was your most important achievement?<br>• What did you find (de)motivating?	• How effective do you think the intervention is?<br>• How do you think the intervention can (not) support

			• Do you think it has made a difference to your abilities, health, or well-being? How?	physiotherapists and patients?<br>• Which functions were most helpful or supportive?
Self-efficacy	The participant's confidence in being able to perform the behaviours required to participate in the intervention	• Confidence<br>• Competences<br>• Usefulness	• To what extent did you feel capable of managing the app and doing the exercises?<br>• How well did the programme match your needs?<br>• What did you do when/if you felt the need for help or support?	• Did you experience any challenges in delivering the intervention?<br>• How prepared and capable did you find the patients and relatives?<br>• How easy will it be for other physiotherapists to take over from you?
Intervention coherence	The extent to which the participant understands the intervention and how it works	• Appropriateness<br>• Relevance<br>• Satisfaction	• How appropriate is it to start exercising at home early after surgery?<br>• Have your experiences in this project influenced your views and expectations of CR?<br>• Would you recommend the app-based programme to others? Why (not)?	• How well can this intervention supplement existing rehabilitation initiatives?<br>• How do you like the idea of it becoming part of an integrated CR offer?<br>• How do you think the intervention can become sustainable for the patients?
