# Supplementary material for: Feasibility of early digital health rehabilitation after cardiac surgery in the elderly: a qualitative study
Source: BMC Health Serv Res. 2024 Jan 22;24:113. doi: 10.1186/s12913-024-10601-3 (PMC10801932; doi:10.1186/s12913-024-10601-3)
Supplement: Supplementary file 3 — Additional file 3. Quotations from patients (P), relatives (R), and physiotherapists (PT) illustrating themes and sub-themes. [file 12913_2024_10601_MOESM3_ESM.pdf]

Additional file 2: Quotations from patients (P), relatives (R), and physiotherapists (PT) illustrating themes and sub-themes

Theme 1: Creating an individual fit by tailoring the intervention	
**1a: Unilateral focus on exercise and physical factors**	
**Patients and relatives**	**Physiotherapists**
"I don't know if you understand if you haven't had your chest cut open yourself…will it last? Has it been sewed properly?" P12<br>"I plan to attend CR. They will address both exercises and the psychological and the whole lot about [that I have had] an anxiety disorder. And in that respect, I think I am a bit alone." P4<br>"It became obvious that he had run into a depression and it turned out that the exercises had faded into the background." R14	"I think for a start if you really wanted to make training that is effective for the patients, then it should be more individualized because it is so different, what is beneficial for the patient." PT
**1b: Acknowledging a demanding start**	
"Then on a Tuesday 'clash', then the house of cards toppled and I thought, 'I really can't handle it and start doing these exercises.' (…) It wasn't nice [to start exercising]. I didn't expect it to be that hard." P6<br>"It was really difficult to start with, and then someone came and 'kicked me off'. More times I was asked, 'Well, Dad, have you done your exercises?' So my children have kept me under surveillance." P13<br>"Someone should take him by the hand, saying, 'Let's practise this.' He is back at a stage where someone should help him to get started because he doesn't have the initiative himself." R14	"Instead of looking at the computer to see how they are doing, we could actually call them and say, 'I see you are doing fine' or 'I see you have not used it for the last couple of days. How are you?'" PT
**1c: Individual adaptions responding to users' preferences and capacities**	
"Most of them [exercises], I could not manage at all, for example, hip raises, they were completely impossible to do." P14<br>"I found the exercises a little too easy." P15<br>"When you have done some of the exercises – one was done on the bed – then I can just lie down for a moment and relax and read a little and then I will do the next [exercise] when it suits me." P3<br>"The idea of getting people to start exercising early is right, it's just about how [it is done]. It probably works well for some and then there are the ones where it doesn't and where personal contact with a physiotherapist, who can help one get started, would have been a better solution." P14	"I don't think it will be defined as strength training. It is not targeted well enough to that...my goal is simply to make them [the patients] as active as possible." PT<br>"Perhaps you should not focus this much on the exercises, but on something like getting out of bed." PT
**Theme 2: Prioritizing communication and collaboration**	
**2a: Sufficient support and follow-up**	
"I did not install it immediately because I did not have the energy or desire…so they called me from out there [the hospital] and asked, 'How are you?' and if I had installed it." P13<br>"The possibility of communicating with a physiotherapist on the other end, I think, was the most important thing I needed about the restrictions. It was comforting that I could write." P4<br>"Dad says he has doubled the exercises, I wonder, 'Are you sure that's clever and a part of the plan?' Perhaps the expectations should have been aligned." (R3) "But she [the physiotherapist] wrote that I was great, then I figured it was all right." P3	"They [patients] get a pre-instruction by a physiotherapist, an anaesthesiologist, a surgeon, a nurse, and a pharmacist…There are so many things going on in their minds, and then we come and tell them, 'By the way you also need to have this [device].' (…) If that happened to me, I would say, 'All right, that goes on the bottom of the pile, I am not listening.'" PT
**2b: Balancing obligations and responsibilities**	
"I wanted to contact the physiotherapist to say that I, unfortunately, had to drop out because having the exercises hanging over my head was feeling like being obligated to do them – when you can't. (...) Then I figured, no, you have to try because it is important to get started." P6<br>"When you sign up for such a project, you also need to take it seriously." P13	"I rather thought about it [the monitoring] as a kind of exercise diary, where we can tick off that they [the patients] have done what they were asked to or if they didn't." PT<br>"Being unable to go and sit by the computer and make the adjustments immediately, that, for me, was a bit of a let-down." PT

“I had to do the exercises together with him and structure the day to avoid becoming the one saying, ‘Now it’s time to exercise’…because then you felt I was on your back.” R12	
**2c: Involvement and roles of relatives**	
“I would have liked to be involved from the beginning when he got it [the equipment] and perhaps being told about the option of doing it [exercise] together, using a schedule, avoiding getting the role of, ‘Now she is on my back again.’” R12<br>“If it had not been for me, he would never have started doing it [exercise], but I think it is burdensome because it is me who swings the whip and that is not very nice. That role, I would rather have been without.” R14	“Sometimes the relatives are already sitting in the room listening and nodding, telling us that they will make sure that it [exercise] happens. This contact I actually think is just as good as handing them a phone to keep active.” PT
**Theme 3: Interacting with the mobile health application**	
**3a: Using technology and monitoring**	
“Suddenly, a message showed up on the cell phone that it needed an update. Then I knew I had to go online and it showed something else. It was very confusing. I really didn’t know what to do, so I turned it off.” P4<br>“It makes a difference that you know [someone is following the efforts]. Well, you do that little extra and are not getting away with it easily.” P15<br>“Then I realised that it was actually really easy [to work the technology].” (P2) “That is good feedback from a man aged 74 who was not raised with it [technology].” R2	“But about the quality of what they did [the exercises], how am I to see the level of improvement to step up to the next level? I think that is difficult when I have not watched them do the exercises.” PT
**3b: Timely delivery and organization of intervention**	
“I would suggest that the patient should get a leaflet for his relatives, instead of sitting there discussing it with a patient who has just had a major surgery.” R4	"We must try to instruct them [the patients] as close as possible to the time of discharge. But close to weekends, we will nearly [always] see them day 1 or 2 [after surgery] and they do not necessarily remember much of what we are telling them.” PT<br>"This individual adaption is really difficult to find time to do at follow-up, and then instruction is more like, 'Do as many as you like.’” PT
**3c: The value of closing an intervention gap**	
“If you are alone…it is a splendid invention.” P7<br>“It is really ideal that it [the intervention] is delivered on a phone and it kind of organizes it to start from the beginning and that worked really well.” P12<br>“I think it works and it is nice with a push. I would not have done it [the exercise] if I had not got it [the equipment] with me [at] home.” P15	“For elderly people with little muscle mass, fatigue is a major factor, so even taking on the task of doing these things [exercises], I think will lift them.” PT<br>“Somewhere along the line, I think it would be just as good when we guide them in the exercises in the department or tell them to go for walks.” PT
